# Morphology of Immunomodulation in Breast Cancer Tumor Draining Lymph Nodes Depends on Stage and Intrinsic Subtype

**DOI:** 10.1038/s41598-018-23629-3

**Published:** 2018-03-28

**Authors:** Maximilian Seidl, Moritz Bader, Astrid Vaihinger, Ulrich F. Wellner, Rumyana Todorova, Bettina Herde, Klaudia Schrenk, Jochen Maurer, Oliver Schilling, Thalia Erbes, Paul Fisch, Jens Pfeiffer, Linda Hoffmann, Kai Franke, Martin Werner, Peter Bronsert

**Affiliations:** 1 Institute for Surgical Pathology, Medical Center – University of Freiburg, Freiburg, Germany; 2 Center for Chronic Immunodeficiency, Medical Center – University of Freiburg, Freiburg, Germany; 3 Comprehensive Cancer Center Freiburg, Medical Center – University of Freiburg, Freiburg, Germany; 4 Division of Cranio-maxillo-facial Surgery, Department of Reconstructive Surgery, University of Basel, Basel, Switzerland; 5 Clinic for Surgery, University Clinic Schleswig-Holstein Campus Lübeck, Lubeck, Germany; 6 Department of Gynecology, RWTH Aachen University Hospital, Aachen, Germany; 7 German Cancer Consortium (DKTK) and Cancer Research Center (DKFZ), Heidelberg, Germany; 8 Institute for Molecular Medicine and Cell Research, University of Freiburg, Freiburg, Germany; 9 BIOSS Centre for Biological Signaling Studies, University of Freiburg, Freiburg, Germany; 10 Department of Oto-Rhino-Laryngology, Medical Center – University of Freiburg, Freiburg, Germany; 11 Department of Trauma, Hand and Reconstructive Surgery Giessen, University Hospital Giessen-Marburg, Giessen, Germany; 12 Department of Obstetrics and Gynecology, Medical Center – University of Freiburg, Freiburg, Germany; 13 Faculty of Medicine, University of Freiburg, Freiburg, Germany; 14 Basler Versicherungen, Basel, Switzerland

## Abstract

Cancer research of immune-modulating mechanisms mainly addresses the role of tumor-infiltrating immune cells. Mechanisms modulating the adaptive immune system at the primary activation site – the draining lymph node (LN) – are less investigated. Here we present tumor-caused histomorphological changes in tumor draining LNs of breast cancer patients, dependent on the localization (sentinel LN vs. non-sentinel LN), the tumor size, the intrinsic subtype and nodal metastatic status. The quantitative morphological study was conducted in breast cancer patients with at least one sentinel LN and no neoadjuvant therapy. All LNs were annotated considering to their topographical location, stained for IgD/H&E, digitized and quantitatively analyzed. In 206 patients, 394 sentinels and 940 non-sentinel LNs were categorized, comprising 40758 follicles and 7074 germinal centers. Subtype specific immunomorphological patterns were detectable: Follicular density was higher in LNs of Her2 enriched hormone receptor positive and triple-negative breast cancers whereas hormone receptor positive breast cancers showed more macrophage infiltrations in the LN cortex. Follicles are rounder in metastatic LNs and non-sentinel LNs. The identified immunomorphological changes reflect different underlying immunomodulations taking place in the tumor-draining LNs and should therefore be considered as possible prognostic and predictive markers for LN metastasis and therapy associated immunomodulation.

## Introduction

LN metastasis is crucial in tumor evolution and associated with an adverse outcome in most solid tumors. LNs are organized compartments (marginal sinus, follicle, germinal center, mantle zone) of lymphoid tissue that is passed through by lymph and blood vessels. The LN compartments can concentrate antigens, help to produce corresponding antibodies, and recruit immune cells^1,2^. Depending on the type and amount of the stimulus these compartments can expand or diminish^3–6^. In cancer, tumor draining lymph vessels and endothelial venules – triggered by the tumor – are forming the pre-metastatic niche in tumor draining LNs^7,8^. Tumor derived soluble factors, debris and cells converge via lymph vessels, into the local draining LNs. These are expected to build the foundation for LN metastasis^9–12^ according to the “seed and soil” hypothesis^13,14^.

The current focus of cancer research in immune-modulating mechanisms mainly addresses the role of tumor-infiltrating immune cells as therapeutic targets (such as the PD1/PDL1 axis)^15,16^, representing the effector site of tumor promoting immunity.

The identification of mechanisms modulating the adaptive immune system at the primary activation site – the draining LN – is fairly out of focus. Data for the amount and spatial allocation of immune cells and their descendants in tumor draining LNs reflecting tumorous immunomodulatory transformations are rare and not matched with tumor infiltrating immune cells^17–21^.

The topographic allocation of the lymph drainage (sentinel/adjacent LNs) in breast cancer represents an ideal model to circumstantiate and trace morphological cancer-based immune modulating effects. In addition, the predictive relevant intrinsic subtyping enables insights into the effects of inter-tumoral heterogeneity on LN compartments.

Our project focused on quantitative histomorphological changes in distinct compartments (follicles, germinal centers with their mantle zone, cortical macrophages) of sentinel and non-sentinel regional LNs of breast cancer patients of different intrinsic subtypes.

## Results

All data supporting the below described findings are available from the corresponding author upon reasonable request.

### Descriptive Statistics

In 206 patients, 394 sentinel, 512 level I and 428 level II and III LNs were categorized for LN circumference (1515.40–170385.30 µm, median 36512.90 µm) and area (76017.10 µm²–338119557.90 µm², median 35422800.90 µm²), follicle circumference (120.40–18511.00 µm, median 844.00 µm) and area (946.10 µm²–6372006.90 µm², median 44695.05 µm²), germinal center circumference (74.20–12241.10 µm, median 554.75 µm) and area (399.40 µm²–2677011.90 µm², median 20370.70 µm²) and–if present – metastases circumference (276.60–566460.00 µm, median 55372.80 µm) and area (2060.00 µm²–269337442.60 µm², median 21580975.30 µm²). Altogether, we measured 40758 follicles and 7074 germinal centers in 1334 LNs. All patients contained follicles, whereas in 20 patients no germinal centers were detectable. Calculations of the following parameters were based on these findings.

### Univariate Analyses Identify Significant Correlations Between Clinicopathological Parameters and LN morphometry

**Figure 1:**
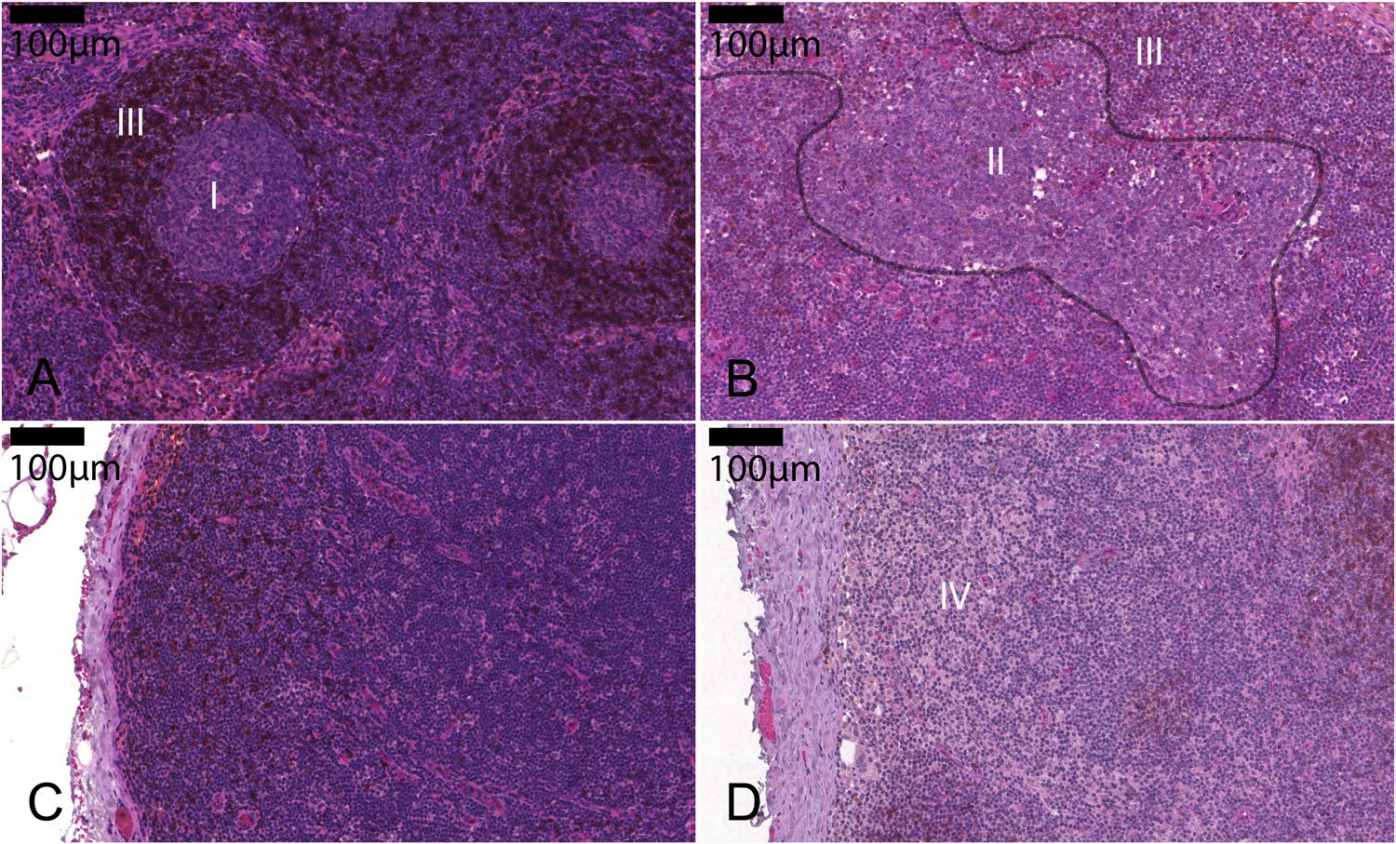
Tumor draining lymph nodes display different immunomorphologies. (**A**) Round germinal center (I) with surrounding IgD+ cells (brown staining) in a well-defined mantle zone (III). (**B**) Almost “naked” non-circular germinal center (II, highlighted by a grey line) with dim mantle zone (III). (**C**) Sparsed IgD+ cells (brown staining) in a lymph node with low follicular density. (**D**) Macrophage infiltration in lymph node cortex (IV). Magnifications as indicated by bar.

Histomorphological parameters were correlated with clinicopathological findings. Follicle and germinal center density, non-circular follicles and germinal centers and cortical macrophage infiltration were analyzed for correlations with age, pT category, nodal status, tumor grading, hormone and Her2 receptor status, and intrinsic subtype, respectively; in all LNs independent of the level (details are listed in Table 1). Patients’ age showed a (non-significant) tendency toward rounder germinal centers (Fig. 1A, exemplary).

**Table 1:** Univariate analyses for all patients.

		Follicle Density	Germinal Center Density	Non-circular Follicles	Non-circular Germinal Centers	Mantle Zone Ratio	Mφ	TILs
Age	min = 29 max = 87 median = 63	p = 0.618 cc = −0.035	p = 0.486 cc = 0.049	p = 0.256 cc = −0.080	p = 0.094 cc = −0.124	p = 0.288 cc = 0.074	p = 0.368 cc = −0.063	p = 0.864 cc = 0.012
pT	T1 (n = 118)	**p < 0.001** cc = 0.260	**p < 0.001** cc = 0.239	p = 0.061 cc = −0.131	**p = 0.033** cc = −0.157	**p = 0.001** cc = 0.228	p = 0.111 cc = −0.111	p = 0.070 cc = 0.127
	T2 (n = 74)							
	T3 (n = 6)							
	T4 (n = 8)							
pN	N− (n = 137)	**p = 0.01** cc = 0.178	**p < 0.001** cc = 0.223	**p = 0.006** cc = −0.189	**p = 0.03** cc = − 0.160	**p = 0.001** cc = 0.225	p = 0.330 cc = −0.068	p = 0.124 cc = 0.108
	N+ (n = 69)							
G	G1 (n = 24)	**p < 0.001** cc = 0.302	**p < 0.001** cc = 0.276	p = 0.062 cc = −0.131	p = 0.884 cc = 0.015	**p = 0.027** cc = 0.154	**p < 0.001** cc = −0.237	**p < 0.001** cc = 0.292
	G2 (n = 113)							
	G3 (n = 69)							
ER	Negative (n = 54)	**p < 0.001** cc = −0.287	**p < 0.001** cc = −0.283	p = 0.799 cc = −0.018	p = 0.913 cc = 0.008	p = 0.059 cc = −0.132	**p < 0.001** cc = 0.423	**p < 0.001** cc = −0.304
	Positive (n = 152)							
PR	Negative (n = 65)	**p < 0.001** cc = −0.325	**p < 0.001** cc = −0.266	p = 0.051 cc = 0.136	p = 0.390 cc = 0.064	p = 0.146 cc = −0.102	**p < 0.001** cc = 0.379	**p < 0.001** cc = −0.269
	Positive (n = 141)							
Her2	Score 0 (n = 67)	p = 0.133 cc = 0.105	p = 0.909 cc = −0.008	p = 0.805 cc = 0.017	p = 0.676 cc = −0.031	p = 0.699 cc = −0.027	p = 0.929 cc = 0.006	p = 0.959 cc = −0.004
	Score 1 (n = 96)							
	Score 2 (n = 16)							
	Score 3 (n = 27)							
Subtype	luminal-A (n = 69)	**p < 0.001** cc = 0.440	**p < 0.001** cc = 0.270	p = 0.443 cc = −0.054	p = 0.650 cc = −0.034	p = 0.143 cc = 0.102	**p < 0.001** cc = −0.264	**p < 0.001** cc = 0.277
	luminal-B (n = 68)							
	luminal-HER (n = 15)							
	HER2-enriched (n = 12)							
	triple-negative (n = 42)							
Follicle Density	n = 206 min = 7.68* max = 480.11* median = 146.96*	cc = 1.000	**p < 0.001** cc = 0.394	p = 0.683 cc = 0.029	p = 0.934 cc = 0.006	p = 0.956 cc = −0.004	**p = 0.003** cc = −0.208	p = 0.10 cc = 0.115
Germinal Center Density	n = 206 min = 0* max = 112.97* median = 19.23*	**p < 0.001** cc = 0.394	cc = 1.000	p = 0.165 cc = −0.097	**p = 0.032** cc = 0.158	**p < 0.001** cc = 0.811	p = 0.167 cc = −0.097	**p = 0.004** cc = 0.199
Non-circular Follicles	n = 206 min = 1.02 max = 2.07 median = 1.20	p = 0.683 cc = 0.029	p = 0.165 cc = − 0.097	cc = 1.000	**p < 0.001** cc = 0.433	**p < 0.001** cc = −0.245	p = 0.329 cc = 0.068	p = 0.947 cc = 0.005
Non-circular Germinal Centers	n = 186 min = 1.01 max = 2.18 median = 1.09	p = 0.934 cc = 0.006	**p = 0.032** cc = 0.158	**p < 0.001** cc = 0.433	cc = 1.000	p = 0.458 cc = 0.055	p = 0.725 cc = −0.026	p = 0.172 cc = 0.101
Mantle Zone Ratio	n = 206 min = 0 max = 0.27 median = 0.02	p = 0.367 cc = 0.063	**p < 0.001** cc = 0.802	p = 0.455 cc = 0.052	**p = 0.001** cc = 0.247	cc = 1.000	p = 0.679 cc = −0.029	**p = 0.014** cc = 0.171
Mφ	n = 206 absent = 168 present = 38	**p = 0.003** cc = −0.208	p = 0.167 cc = − 0.097	p = 0.329 cc = 0.068	p = 0.725 cc = −0.026	p = 0.766 cc = 0.021	cc = 1.000	**p = 0.018** cc = −0.164
TILs	N = 206 Absent = 134 Present = 72	**p = 0.10** cc = 0.115	**p = 0.004** cc = 0.199	p = 0.947 cc = 0.005	p = 0.172 cc = 0.101	**p = 0.014** cc = 0.171	**p = 0.018** cc = −0.164	cc = 1.000

Spearman Rho correlation coefficient (cc) calculation was performed for all items (p value is provided over the cc value). Abbreviations: pT: T-category of the primary tumor; pN: N-category of the primary tumor; G: tumor grading according to Elston and Ellis^66^; ER: estrogen receptor protein positivity as defined by >1% of tumor cell nuclear positivity in immunohistochemical stainings; PR: progesterone receptor protein positivity as defined by >1% of tumor cell nuclear positivity in immunohistochemical stainings; Her2: human epidermal growth factor receptor type 2 positivity according to Wolff *et al*.^68^; follicle density and germinal center density were calculated by number of follicles or germinal centers over lymph node area; calculation of non-circular follicles and germinal centers is described in the methods part; mantle zone ratio was calculated as area ratio of each germinal center over the corresponding follicle; Mφ: cortical macrophage infiltrations as defined in the methods part; TIL: tumor infiltrating lymphocytes as defined in the methods part. Values marked with asterisk are multiplied with 10^−8^.

A higher pT-category was accompanied with a significant (p < 0.05) higher follicular and germinal center density as well as smaller mantle zones (Fig. 1A,C, exemplary). On the other hand, a lower pT-category attended significantly more non-circular germinal centers (Fig. 1B, exemplary) and larger mantle zones (represented by a lower mantle zone ratio). Statistical trends (p > 0.05 and <0.15) were observed between pT-category and non-circular follicles as well as cortical macrophage infiltrations (Fig. 1D, exemplary).

Considering the nodal status, an advanced pN-category showed a significantly higher follicular and germinal center density. Higher pN-category showed significantly rounder follicles, rounder germinal centers and smaller mantle zones. Tumor grading/differentiation correlated significantly positive with tumor infiltrating lymphocytes (TILs), an elevated follicular and germinal center density and negative with cortical macrophage infiltration. Statistical trends were observed for non-circular follicles.

Hormone receptor expression for estrogen and progesterone correlated significantly positive with cortical macrophage infiltration and negative with TILs, follicular and germinal center density. Additionally, hormone receptor positive cases tend to have larger mantle zones, progesterone receptor expression revealed a positive statistical trend for non-circular follicles. Considering the Her2 status, a positive statistical trend for follicle density was observed.

### Linear Regression Model Analyses Identify Independent Correlations Between Clinicopathologic Parameters and Morphometrical Data

Univariate significant correlations and trends (Table 1) were included into a linear regression for multivariate analysis (detailed values are enlisted in Table 2). Considering clinicopathologic parameters, linear regression analyses revealed independent correlations between follicle density and the intrinsic subtype, between tumor grading and follicle roundness as well as between follicle and germinal center roundness and mantle zone ratio. Furthermore, independent correlations between follicle density and germinal center density but also between follicle roundness and germinal center roundness were detected.

**Table 2:** Multivariate linear regression model overall patients with a significant p-value (p < 0.05) or statistical trend (p > 0.05 and <0.150) in the univariate analyses using Spearman Rho correlation coefficient (Table 1).

	Follicle Density	Germinal Center Density	Non-circular Follicles	Non-circular Germinal Centers	Mantle Zone Ratio	Mφ	TILs
Age	n.i.	n.i.	n.i.	n.s.	n.i.	n.i.	n.i.
pT	n.s.	n.s.	n.s.	n.s.	n.s.	n.s.	n.s.
pN	n.s.	n.s.	n.s.	n.s.	n.s.	n.i.	n.s.
G	n.s.	n.s.	**p = 0.045**	n.i.	n.s.	n.s.	n.s.
ER	n.s.	n.s.	n.i.	n.i.	n.s.	n.s.	n.s.
PR	n.s.	n.s.	n.s.	n.i.	n.s.	n.s.	n.s.
Her2	n.i.	n.i.	n.i.	n.i.	n.i.	n.i.	n.i.
Subtype	**p = 0.001**	n.s.	n.i.	n.i.	n.s.	n.s.	n.s.
Follicle Density	n.i.	n.s.	n.i.	n.i.	n.i.	n.s.	n.s.
Germinal Center Density	**p < 0.001**	n.i.	n.i.	n.s.	**p < 0.001**	n.i.	n.s.
Non-circular Follicles	n.i.	n.i.	n.i.	n.s.	**p = 0.005**	n.i.	n.i.
Non-circular Germinal Center	n.i.	n.s.	**p < 0.001**	n.i.	n.i	n.i.	n.i.
Mantle Zone Ratio	n.i.	n.s.	n.i.	n.s.	n.i.	n.i.	n.s.
Mφ	n.s.	n.i.	n.i.	n.i.	n.i.	n.i.	n.i.
TILs	n.s.	n.s.	n.i.	n.i.	n.s.	n.s.	n.i.

Abbreviations: pT: T-category of the primary tumor; pN: N-category of the primary tumor; G: tumor grading according to Elston and Ellis^66^; ER: estrogen receptor protein positivity as defined by >1% of tumor cell nuclear positivity in immunohistochemical stainings; PR: progesterone receptor protein positivity as defined by >1% of tumor cell nuclear positivity in immunohistochemical stainings; Her2: human epidermal growth factor receptor type 2 positivity according to Wolff *et al*.^68^; follicle density and germinal center density were calculated by number of follicles or germinal centers over lymph node area; calculation of non-circular follicles and germinal centers is described in the methods part; mantle zone ratio was calculated as area ratio of each germinal center over the corresponding follicle; Mφ: cortical macrophage infiltrations as defined in the methods part; TIL: tumor infiltrating lymphocytes as defined in the methods part.

### Subtype-specific Analyses Reveal Distinctive Immunomorphological Patterns in the LNs

**Figure 2:**
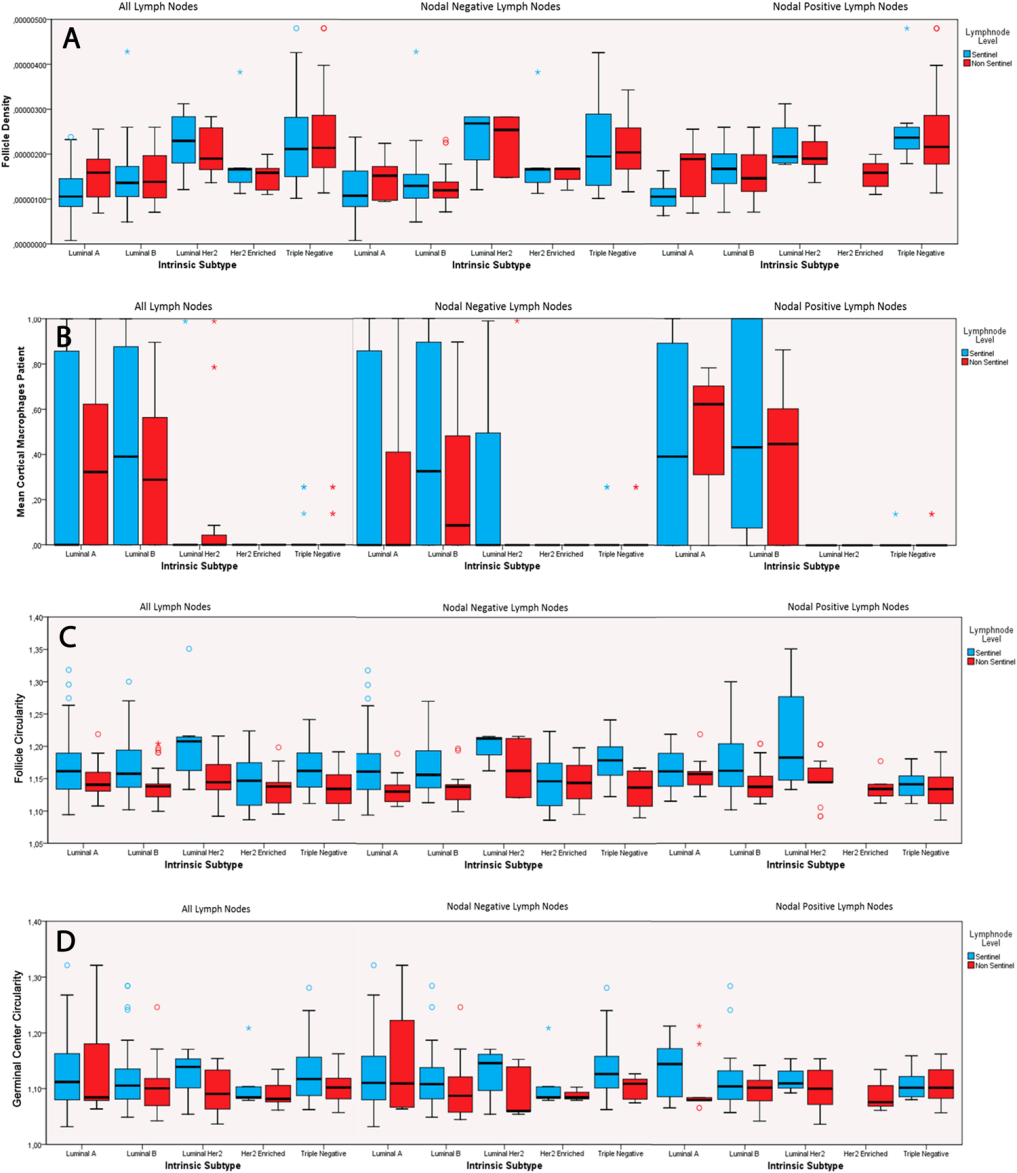
Intrinsic subtypes show differences in follicular densities (**A**), cortical macrophage infiltrations (**B**), follicular and germinal center shape (**C**,**D**); value = 1 means a perfect circle, higher values mean more irregular structures). Boxplots show median values, 66% percentiles and standard deviations, respectively. Absolute numbers of patients are highlighted in Table 4. Data underlying B was dichotomous as cortical macrophages present (=1) or absent (=0), leading to values between 0 and 1, with a possible median value = 0.

**Figure 3:**
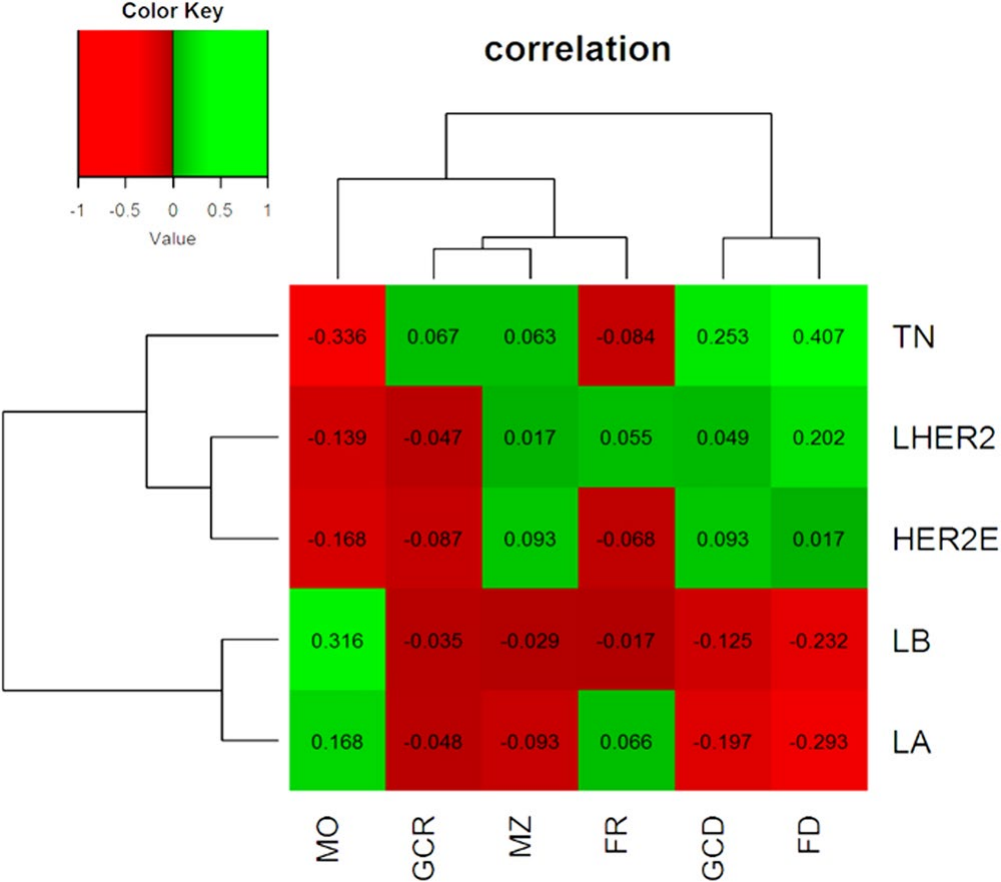
Heatmap of correlation coefficients shows subtype specific immunomorphological patterns. Parameters on the Y-axis: cortical macrophage infiltration (MO), non-circular germinal centers (GCR), mantle zone (MZ), non-circular follicles (FR), germinal center density (GCD) and follicle density (FD). Intrinsic subtypes are shown on the Y-axis: triple-negatives (TN), luminal-Her2 (LHER2), Her2-enriched (HER2E), luminal-B (LB) and luminal-A (LA).

For subtype-specific analysis, all morphometric data (follicular and germinal center density, non-circular follicles and germinal centers, mantle zone ratio, cortical macrophages and tumor infiltrating lymphocytes) were included and tested for each intrinsic subtype separately. Follicle density correlated significantly positive (p < 0.05) with the luminal-Her2 and triple-negative subtype and significantly negative (p < 0.05) with the luminal-A and -B subtypes, respectively. Germinal center density correlated positive with the triple-negative and negative with the luminal-A and -B subtypes. Cortical macrophages and luminal-A and -B subtype demonstrated positive correlations, whereas the luminal-Her2, the Her2-enriched and the triple-negative subtypes correlated significantly negative with the amount of cortical macrophages. Regarding the effector site, TILs correlated negatively with the luminal-A and -B but positively with the triple-negative subtypes, respectively. Statistical trends were observed between germinal center density and Her2-enriched tumors, follicle roundness and triple-negative subtype and mantle zone with luminal-A and Her2-enriched tumors. Further results are presented in Table 3, Fig. 2 (absolute numbers of patients are highlighted in Table 4), and visualized in a heatmap (Fig. 3).

**Table 3:** Subtype stratified univariate analyses for all patients.

	Follicle Density	Germinal Center Density	Non-circular Follicles	Non-circular Germinal Centers	Mantle Zone Ratio	Mφ	TILs
Luminal-A vs. all other subtypes	p = < **0.001** cc = −0.293	p = **0.001** cc = −0.197	p = 0.252 cc = 0.066	p < 0.436 cc = −0.048	p = 0.106 cc = −0.093	**p = 0.003** cc = 0.168	**p = 0.043** cc = −0.041
Luminal-B vs. all other subtypes	**p = < 0.001** cc = −0.232	**p = 0.028** cc = −0.125	p < 0.765 cc = −0.017	p = 0.565 cc = −0.035	p = 0.608 cc = −0.029	**p = < 0.001** cc = 0.316	**p = 0.022** cc −0.160
Luminal-Her2 vs. all other subtypes	**p = < 0.001** cc = 0.202	p = 0.397 cc = 0.049	p = 0.334 cc = 0.055	p = 0.446 cc = −0.047	p = 0.768 cc = 0.017	**p = 0.015** cc = −0.139	p = 0.672 cc = −0.030
Her2-enriched vs. all other subtypes	p = 0.763 cc = 0.017	p = 0.106 cc = 0.093	p = 0.233 cc = −0.068	p = 0.158 cc = −0.087	p = 0.104 cc = 0.093	**p = 0.003** cc = −0.168	p = 0.262 cc = 0.078
Triple-negative vs. all other subtypes	**p = < 0.001** cc = 0.407	**p = < 0.001** cc = 0.253	p = 0.145 cc = −0.084	p = 0.273 cc = 0.067	p = 0.271 cc = 0.063	**p = < 0.001** cc = −0.336	**p < 0.001** cc = 0.286

Spearman Rho correlation coefficient (cc) calculation was performed for all items (p value is provided over the cc value). Follicle density and germinal center density were calculated by number of follicles or germinal centers over lymph node area; calculation of non-circular follicles and germinal centers is described in the methods part; mantle zone ratio was calculated as area ratio of each germinal center over the corresponding follicle; Mφ: cortical macrophage infiltrations as defined in the methods part; TIL: tumor infiltrating lymphocytes as defined in the methods part.

**Table 4:** Absolute numbers of patients with regard to the lymphnode level (sentinel or non-sentinel) and immunohistological subtype.

	All		Nodal Negative Lymph Nodes		Nodal Positive Lymph Nodes	
Lymphnode Level	Sentinel	Non Sentinel	Sentinel	Non Sentinel	Sentinel	Non Sentinel
Luminal A	60	26	50	15	10	11
Luminal B	59	40	41	12	18	28
Luminal Her2	8	19	4	5	4	14
Her2 enriched	8	10	7	2	1	8
Triple negative	26	50	19	16	7	34
Total	161	145	121	50	40	95

### Follicular Density, Follicular Shape, and Cortical Macrophage Infiltrations Differ Between Draining LN Stations and Intrinsic Subtypes

For the determination of subtype-specific immunomorphological changes in the position-dependent LN draining stations (sentinel LN vs level I-III) morphological differences in sentinel *vs* non-sentinel LNs were evaluated over all subtypes and for each subtype separately (Table 5, Fig. 2A–D).

**Table 5:** Level stratified univariate analyses in each subtype for all patients.

	Follicle Density	Germinal Center Density	Non-circular Follicles	Non-circular Germinal Center	Mantle Zone Ratio	Mφ
Sentinel vs Level I-III (all subtypes)	**p = 0.001** cc = 0.105	p = 0.077 cc = 0.101	**p < 0.001** cc = −0.409	**p < 0.001** cc = −0.333	p = 0.78 cc = 0.101	**p < 0.001** cc = −0.216
Sentinel vs Level I-III luminal-A	p = 0.083 cc = 0.203	p = 0.744 cc = 0.039	**p = 0.02** cc = −0.27	**p = 0.013** cc = −0.312	p = 0.449 cc = 0.089	p = 0.795 cc = 0.031
Sentinel vs Level I-III luminal-B	p = 0.451 cc = 0.08	p = 0.978 cc = −0.003	**p < 0.001** cc = −0.508	**p = 0.008** cc = −0.299	p = 0.679 cc = 0.044	**p = 0.019** cc = −0.246
Sentinel vs Level I-III luminal-HER2	p = 0.569 cc = −0.115	p = 0.467 cc = 0.146	**p = 0.004** cc = −0.531	p = 0.133 cc = −0.331	p = 0.099 cc = 0.324	p = 0.813 cc = −0.048
Sentinel vs Level I-III HER2-enriched	p = 0.609 cc = −0.129	p = 0.956 cc = −0.011	p = 0.494 cc = −0.172	p = 0.102 cc = −0.424	p = 0.702 cc = 0.097	No cortical macrophages present
Sentinel vs Level I-III triple-negative	p = 0.565 cc = 0.067	p = 0.737 cc = −0.039	**p < 0.001** cc = −0.508	**p < 0.001** cc = −0.475	p = 0.641 cc = −0.054	p = 0.987 cc = 0.002

Spearman Rho correlation coefficient (cc) calculation was performed for all items (p value is provided over the cc value). Follicle density and germinal center density were calculated by number of follicles or germinal centers over lymph node area; calculation of non-circular follicles and germinal centers is described in the methods part; mantle zone ratio was calculated as area ratio of each germinal center over the corresponding follicle; Mφ: cortical macrophage infiltrations as defined in the methods part.

Considering all subtypes, follicle density is lower in sentinel vs non-sentinel LNs (positive correlation with higher LN level, Table 5), whereas non-sentinel LNs displayed rounder follicles and germinal centers (negative correlations for non-circular follicles and germinal centers and higher LN level). Cortical macrophage infiltrations were found to be lower in non-sentinel LNs compared to Sentinel LNs.

Specific analyses for each of the intrinsic subtypes showed luminal-A, -B and triple-negative tumors to have significantly rounder follicles and germinal centers in the non-sentinel LNs (negative correlations between follicle and germinal center un-roundness in sentinel vs level I-III LNs, Table 5). The luminal-B subtype demonstrated more cortical macrophages in sentinel LNs compared to non-sentinel LNs (negative correlation between cortical macrophage infiltration and higher LN level, Table 5). The luminal-Her2 subtype also displayed rounder follicles in non-sentinel LNs and a statistical trend for rounder germinal centers (significant negative correlation with non-circular follicles and higher LN levels and statistical trend for negative correlation between non-circular germinal centers and higher LN levels; Table 5).

Further statistical trends (p > 0.05 and < 0.15) were observed between luminal-A subtype and follicle density, luminal-Her2 as well as Her2-enriched subtype and germinal center roundness.

## Discussion

Our data demonstrate the immune-modulating effects in tumor draining LNs. The study comprised 206 breast cancer patients with 1334 defined LN stations manually annotated, measured, and analyzed for the presence of macrophages, follicles, germinal centers and LN metastasis. Data was generated with a very high granularity. It can be traced back down to the single follicle of each LN of each patient, and was matched with the primary tumor as immunological effector site. To our knowledge, such a detailed analysis has not been compiled before.

Immune-modulated histomorphological changes in LNs can be summarized into subtype-independent groups (pT and pN) and subtype-dependent groups (tumor grade, estrogen and progesterone receptor protein expression).

Advanced tumor growth, i.e. the pT category, revealed a significant positive correlation with follicular and germinal center density, germinal center roundness, and smaller mantle zones. Using an example, a high pT stage is accompanied by an elevated follicular and germinal center density with quantitatively more round germinal centers and with smaller mantle zones.

On the other hand, hormone receptors as the aforementioned tumor-intrinsic factors demonstrated a significant negative correlation for follicle and germinal center density. Furthermore, they are affecting the presence of tumor infiltrating lymphocytes within the tumor and the cortical macrophages within the LNs: A decrease of the estrogen receptor expression was accompanied with a higher number of tumor infiltrating lymphocytes within the tumor and with a higher follicle and germinal center density. A decrease of the estrogen receptor protein was also accompanied by a lower number of cortical macrophages within the draining LN. Notably, all observed changes were not affected by patients’ age.

There are published findings of increased and decreased dendritic cell infiltrations in tumor draining LNs: Laguens *et al*. indicated that the LNs which are draining solid tumors present decreased dendritic cells compared with controls. Their observations are based on a cohort of various tumor entities (larynx (n = 19), breast (n = 18), colon (n = 4) and lung carcinoma (n = 6)) and did not refer back to entity and/or subtype specific characteristics^22^. Laguens *et al*. correlated their data with a pool of LN specimens from cancer-free patients while we performed a meta-analysis over all LNs due to the lack of LNs from patients without immunomodulating diseases (“silent LNs”) - a condition virtually absent in regional LN resection specimens. Lee *et al*. gave insights in the T-cell compartment as well as CD1a+ dendritic cell infiltrations of tumor draining LNs in breast cancer^18^. The latter were found to be increased in metastasis-free tumor draining axillary LNs of nodal positive cases and even those cases without any metastasis. Our results support both studies. Furthermore, taking into account the whole group of macrophages, we were able to expand the perspective to each intrinsic subtype for the whole compartment of macrophages outside sinuses infiltrating the LN cortex, indicative for their potential of antigen presentation^23,24^. Higher cortical macrophage infiltrations were strongly associated with the intrinsic luminal-A and -B subtypes (Table 1., correlation subtype and macrophages p < 0.001, correlation ER/PR and macrophages p < 0.001) and correlated with lower follicular densities in their draining LNs compared to higher follicular densities and lower cortical macrophage infiltrations in the Her2-enriched and the triple-negative subtype. This distribution of macrophages in LNs of the luminal-A and -B subtypes weakly resembles the pattern in dermatopathic lymphadenopathy, which has been described in tumor draining LNs of breast cancer as early as 1975^25^. We did not find significant correlations between increased cortical macrophages and nodal status (Table 1., correlation pN and macrophages p = 0.330). Separated analysis of nodal positive and nodal negative cases showed cortical macrophages being present in both states and therefore we do not believe them to be protective – at least regarding the luminal subtypes. There are interesting functional data from mouse models claiming migratory dendritic cells from the skin mediate downregulation of cellular immune responses after protein vaccine^26^, which might be a mechanism behind local immune tolerance in the luminal subtypes.

Mentioning another form of LN’s involvement by macrophages, sarcoid like lesions were described as pathomorphological findings in tumor draining LNs of different malignancies, including one case of breast cancer More than 60 years ago^17^. However, granulomas/sarcoid-like lesions were rare findings in our cohort, being present in only 4 cases (data not shown). The observation of higher cortical macrophage infiltrations inversely correlates with follicular density (highlighted by IgD staining) in our cohort, which possibly hints towards opposing mechanisms influencing the lymph nodes’ population. Murine data showed IgD positive naïve B-cells (and memory B-cells) populate lymph node follicles via the CD62L/CCR7/CXCR4 mediated passage of high endothelial venules and then further through CXCR5 signaling^27–29^. The egress is dependent on a sphingosine 1-phosphate gradient, with high levels of sphingosine 1-phosphate in blood and lymph vessels^30,31^. Therefore, lowered B-cell immigration or increased egress would be dependent on the modulation of the aforementioned receptors and factors. Whether it might be beneficial to antagonize the egress of lymphocytes (e.g. with FTY720/Fingolimod)^32^ in draining lymph nodes of hormone receptor positive breast cancers remains to be experimentally proven e.g. in animal models or by studying the draining lymph node histology under conditions of therapeutic hormone ablation. On the other hand, macrophages and their derivatives (especially dendritic cells) are – beyond their key role as antigen presenting cells – strong regulators of the amount and function of high endothelial venules^33–35^. However, differently polarized macrophages (M1 vs. M2) may differently modulate the vasculature also in lymph nodes so further characterization of the macrophage populations in the tumor draining lymph nodes is necessary prior to experimentally address the potential role of e.g. antiangiogenic immunomodulatory drugs^36,37^. Another possibility could be the direct depletion of macrophages, which has experimentally been undertaken in tumors^38,39^. However, this could affect also antitumoral macrophages, leading to lower survival rates^40^. An elegant way to recruit macrophages for an antitumoral response starts at its very beginning through activating pattern recognition receptors as e.g. TLR9^40^. Those therapies may also have an impact on the composition of macrophages and lymphocytes in the tumor draining lymph nodes, which yet remains to be characterized.

Setiadi *et al*. analyzed the spatial distribution of B- and T-cells in sentinel and non-sentinel LNs of 15 breast cancer (without referencing UICC classification and intrinsic subtypes) patients and identified B-cells to be more clustered than T-cells in the tumor draining LNs compared to controls^19^. This clustering was more prominent in axillary LNs with metastasis, followed by metastasis-free axillary LNs, followed by sentinel LNs with metastasis. In accordance with this finding, our data showed the follicular density being higher in nodal positive cases (Table 1) and in non-sentinel LNs (Table 4) in the univariate analysis. We identified lower follicular densities in LNs from the luminal-A and -B subtype. In contrast, the luminal-Her2 and triple-negative subtypes demonstrated higher follicular densities. Of note, follicular densities between sentinel and non-sentinel LNs were comparable in all groups except the luminal-A subtype with trend towards lower follicular density in the sentinel LN (Tables 3 and 5, Fig. 2A). These observations are supported by data from oral squamous cell carcinoma, with higher reactive follicle numbers being associated with better overall survival^21,41^. In our study, the number of disease-associated deaths was too low to identify independent effects on the patients’ overall survival. Only pT- category and intrinsic subtype were significant independent prognosticators in multivariate analysis. However, the luminal-Her2 and the triple-negative subtype – both showed higher follicular densities – are more aggressive cancers^42^. In comparison with the aforementioned head and neck LN findings^21,22^, our data emphasizes that specific tumor-dependent protective immunomorphological patterns are not necessarily transferable onto other tumor entities.

Comparing tumor draining LNs and the primary tumor site, the triple-negative subtype resembles the luminal-Her2 subtype in follicular density and shape, whereas tumor infiltrating lymphocytes are a dominant feature of only the triple-negative subtype (Table 3). To our knowledge, there are currently no available comparative results of immunomodulatory therapies in triple-negative and luminal-Her2 breast cancer to postulate whether there is a similar (mirroring similar LN findings) or different therapeutic response (mirroring different TIL infiltration). This information may perhaps be extracted from ongoing and planned future studies summarized by Spellman and Tang^43^.

Little is known about the shape of follicles and germinal centers in tumor draining LNs. Berlinger *et al*. described reactive follicles with germinal centers tending to “fuse” in tumor draining LNs of head and neck squamous cell carcinoma^41^. Regarding the microphotographies of this paper, these “fused” germinal centers are the same we classified as non-circular in this actual and a previous study^44^. Non-circular germinal centers can be found in infectious lymphoadenopathies as toxoplasmosis, EBV and HIV^45–48^. Further associations exist with Common Variable Immunodeficiency (CVID) – especially those of the Freiburg class Ia group with stronger immune dysregulation^44^, and angioimmunoblastic T-cell lymphoma^49–51^. The functional background shaping this germinal center morphology remains elusive but there is experimental data indicating a role of higher interferon gamma levels in CVID patients^52^.

Follicular helper T cells (Tfh) play an essential role in the germinal center formation, maintenance and output, e.g. through mediating the class switch recombination of germinal center B-cells^53^. Tfh express PD1 and are pronounced in the light zone of the germinal centers^54,55^. Data in mice indicate PD1 signaling being important for the germinal center output of long lived, class switched plasma cells as well as immunoglobulin levels and affinities^56–59^. However, results are heterogenous as different costimulatory molecules and points in time were observed in the different groups. The impact of PD1 inhibitor therapy on germinal center morphology has not been described to date. Cytotoxic T-lymphocyte-associated Protein 4 (CTLA4) as a further therapeutic target of immune checkpoint inhibition plays an important role for Tfh function and the germinal center’s shape^60^. CTLA4 antagonization or deficiency is associated with large, ill-defined germinal centers and lowered serum immunoglobulin levels^60,61^.

Evaluating the germinal center size and shape may therefore be a potential predictive marker to better define patients who would benefit from therapies targeting immune checkpoints, as intra- and peritumoral genetic and/or immunohistochemical markers alone are not sufficient to predict therapeutic outcome yet^62–64^. Based on our findings and the published data discussed, we hypothesize an increased benefit from immune checkpoint inhibition in patients with high densities of germinal centers with a round shape in their tumor draining lymph nodes.

To conclude, we show that breast cancer subtype-specific immunomorphological changes reflecting different underlying immunomodulations take place in the tumor draining LNs. These immunomorphological changes may help to study the patients’ immune resources beyond the primarius’ effector site and may help to guide patient selection to immunomodulatory drug treatment. Further studies in tumor draining LNs are necessary to characterize the functional background of those different immunomorphologies for their therapeutic targeting in the future.

## Material and Methods

### Patients and cohort

After ethics approval by local authorities (Ethics Committee University Medical Center Freiburg; voted application number 10011/16) according to the Helsinki declaration, matching tumor and regional LN samples of 206 patients (operated between 2003 to 2013) were retrieved from the local biobank. Surgery for primary breast cancer and corresponding LNs was performed at the Department of Obstetrics and Gynecology at the University Medical Center Freiburg. Inclusion criteria were existing sentinel LN, absence of neoadjuvant therapy and absence of anti-hormonal treatment prior to resection. All surgically removed LN samples were assigned according to their LN level (sentinel, level I, level II, and level III). Of note, all tumors were histologically and immunohistologically classified according to their WHO subtype^65^, tumor grading (Elston and Ellis)^66^, the expression of estrogen and progesterone receptor proteins^67^, MIB-1 based proliferation index, and human epidermal growth factor receptor 2 (Her2neu)^68^ expression. In case of Her2neu score 2+, chromogenic *in situ* hybridization was performed. Subsequently, all tumors were classified with respect to their molecular subtype (luminal-A, luminal-B, luminal-Her2, Her2-enriched, and triple-negative) according to the 2011 St. Gallen guidelines^69^. Patients’ characteristics are listed in Table 1.

### H&E/IgD Staining

For the observation of B-cell follicles, mantle zones, germinal centers, and LN morphology, H&E/IgD “double” staining was performed. Slides of 2 µm were dried at 58 °C overnight and deparaffinized in xylene and decreasing ethanol concentrations. Heat-mediated epitope retrieval was performed for 20 minutes at pH 6.1 and 95 °C. Slides were stained with hematoxylin before further proceeding with IgD staining. IgD immunohistochemistry was performed on an automated staining system using a horseradish peroxidase catalyzed brown chromogen reaction according to the manufacturer’s guidelines (antibody: polyclonal rabbit anti-human IgD, IS51730-2, ready to use, Dako, Hamburg, Germany; staining: Autostainer Plus, Dako). The slide was then stained with Eosin Y (Leica Biosystems, Nussloch, Germany) and covered with a coverslip.

### Estrogen, Progesterone, Her2 and Ki67 Staining

The intrinsic subtype was immunohistologically identified via ready-to-use antibodies for the estrogen-receptor protein (monoclonal rabbit anti-human estrogen receptor α, clone EP1, code IR084, Dako) progesterone-receptor protein (monoclonal mouse anti-human progesterone receptor, clone PgR 636, code IR068, Dako), Her2 (polyclonal rabbit anti-human c-erbB-2 oncoprotein, code A0485, Dako) and Ki-67 (monoclonal mouse anti-human Ki-67 antigen, clone MIB-1, code IR626, Dako). For the horse-radish based peroxidase detection, EnVision Flex Peroxidase-Blocking Reagent (DAKO, SM801), EnVision Flex+ Rabbit (LINKER) (DAKO, K8019) or EnVision Flex+ Mouse (LINKER) (DAKO, K8021) and EnVision Flex/HRP (DAKO, SM802) were used. Counterstaining was performed with hematoxylin before adding the coverslip.

As an internal positive control, patient-derived, non-neoplastic mammary glands were used for ER, PR and Ki-67 (nuclear staining). For Her2, tissue specimens from Her2 positive breast cancer patients (score 3 according to Wolff *et al*.^68^) were included for every Her2 staining session as external positive control.

### Slide Scanning and Evaluation of LNs

**Figure 4:**
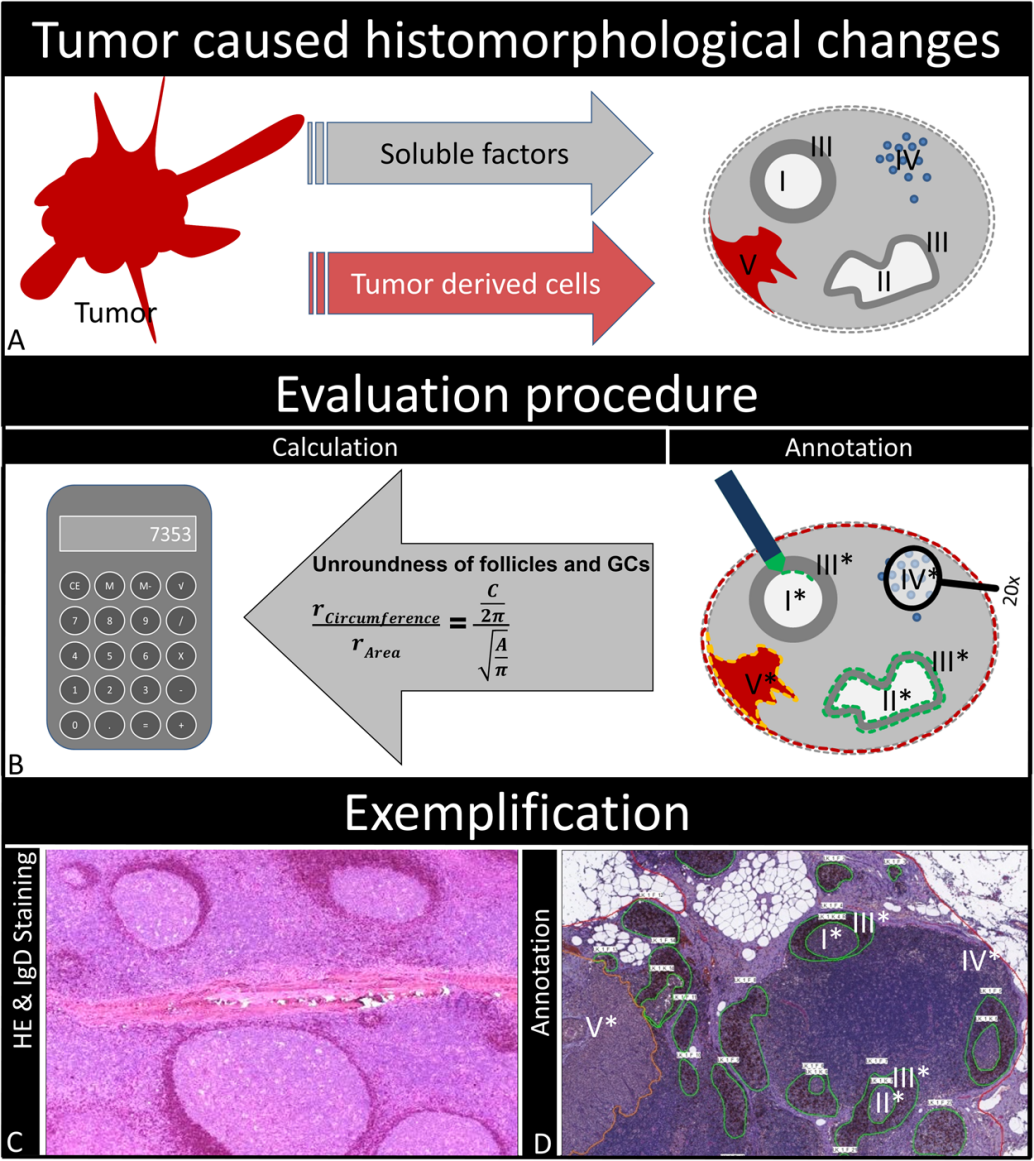
Workflow. (**A**) Tumor and matching lymph nodes were included and analyzed based on anatomic immunological structures: Germinal centers (I round, II non-circular), follicles and germinal center mantle zones (III), cortical macrophage infiltrations (IV) and lymph node metastasis (V). (**B**) Structures were annotated for further calculations as area densities and roundness. (**C**) H&E/IgD stainings were performed on all lymph nodess to highlight the structures. (**D**) Example of fully annotated H&E stained lymph node section.

Morphometrical analysis was performed on digitized slides (detailed workflow is presented in Fig. 4). All slides were annotated with a unique identifier, scanned with a 20 × objective (Mirax Scan Pannoramic Scanner, 3DHistec, Hungary) and analyzed using the manufacturer’s proprietary software (Pannoramic Viewer, version 1.15.4, 3DHistec). B-cell follicles and matching germinal centers, LNs and (if present) metastases were individually annotated and parsed for each LN separately using the software’s annotation function. Circumferences and areas of the named structures were exported as.csv file (Pannoramic Viewer, version 1.15.4, 3DHistec) for further quantitative analysis: number and area of LNs and metastases as well as number, area, circumference and circularity of follicles and matching germinal centers. Roundness of germinal centers and follicles was calculated as previously described^44^. Briefly, radii of follicle and germinal center were calculated from circumference and area and put into ratio. A ratio of one represents a perfect circle – in our case a round follicle and/or germinal center – a ratio >1 represents a non-circular structure – in our case a non-circular follicle and/or germinal center. Mantle zones were calculated as ratio of germinal center area divided through follicle area. LN cortical macrophage infiltration was measured semi-quantitatively in each LN as infiltration of follicular and inter-follicular areas (sinuses were excluded): three spots of macrophage-rich cortical infiltration (diameter of 1 HPF, digital 40x magnification on 17″ screen) or one spot in a 20x field diameter. Cortical macrophages were graded per LN as either present (=1) or absent (=0).

Follicular and germinal center density was calculated as numbers of follicles and germinal centers divided by the area of the corresponding LN.

Mantle zone was calculated by dividing germinal center area by follicle area.

Regarding primary tumors, lymphocytic infiltrations in tumors were measured semiquantitatively on H&E sections with conventional microscopy. Tumors were considered “lymphocyte rich” if coherent lymphocytic infiltrates were visible in 20x field of view (microscope: Leica DM 2500, Wetzlar, Germany).

All annotations were continuously reviewed and discussed by two experienced pathologists until consensus was reached. Until consensus, the pathologists were blinded for all clinicopathological parameters.

### Statistical Analysis

For descriptive statistics and statistical testing, SPSS version 21 (IBM SPSS Inc, Chicago, IL, U.S.A.) was used. Scale variables were expressed as median and range, categorical parameters by cross tabulation and percentages. For statistical testing, two-sided Chi-squared, Mann-Whitney, Spearman rank correlation and logistic regression models were performed. A significance level of 5% was chosen. Heatmap and Clustering were performed with the R package gplots 3.31.
